# Hydroxytyrosol Promotes Proliferation of Human Schwann Cells: An In Vitro Study

**DOI:** 10.3390/ijerph17124404

**Published:** 2020-06-19

**Authors:** Khidhir Kamil, Muhammad Dain Yazid, Ruszymah Bt Hj Idrus, Jaya Kumar

**Affiliations:** 1 Department of Physiology, Faculty of Medicine, Universiti Kebangsaan Malaysia Medical Centre, Kuala Lumpur 56000, Malaysia; khidhirkamil@gmail.com (K.K.); ruszyidrus@gmail.com (R.B.H.I.); 2 Tissue Engineering Centre, Faculty of Medicine, Universiti Kebangsaan Malaysia Medical Centre, Kuala Lumpur 56000, Malaysia; dain@ukm.edu.my

**Keywords:** hydroxytyrosol, Schwann cell, peripheral nerve injury, olive, nerve regeneration, proliferation, p75 NGFR, GFAP, cell cycle

## Abstract

Recent advances in phytomedicine have explored some potential candidates for nerve regeneration, including hydroxytyrosol (HT). This study was undertaken to explore the potential effects of HT on human Schwann cells’ proliferation. **Methods:** The primary human Schwann cell (hSC) was characterized, and the proliferation rate of hSC supplemented with various concentrations of HT was determined via 3-(4,5-dimethylthiazol-2-yl)-2,5-diphenyltetrazolium bromide (MTT) assay. Cell cycle analysis and protein expression of glial fibrillary acidic protein (GFAP) and p75 nerve growth factor receptor (p75 NGFR) were evaluated via the immunofluorescence technique. **Results:** In vitro culture of hSCs revealed spindle-like, bipolar morphology with the expression of specific markers of hSC. Hydroxytyrosol at 10 and 20 ng/mL significantly increased the proliferation of hSCs by 30.12 ± 5.9% and 47.8 ± 6.7% compared to control (*p* < 0.05). Cell cycle analysis showed that HT-treated hSCs have a higher proliferation index (16.2 ± 0.2%) than the control (12.4 ± 0.4%) (*p* < 0.01). In addition, HT significantly increased the protein expression of GFAP and p75NGFR (*p* < 0.05). **Conclusion:** HT stimulates the proliferation of hSCs in vitro, indicated by a significant increase in the hSC proliferation index and protein expression of hSCs’ proliferation markers, namely p75 NGFR and GFAP.

## 1. Introduction

Recent advances in phytomedicine have reported various health-promoting effects of olive oil and its extracts [1,2,3,4]. Olive oil contains numerous phytochemicals/phenolic compounds such as oleuropein, tyrosol, and hydroxytyrosol that are known to have potent antioxidant and cardio-neuroprotective effects [5,6,7,8,9]. The neuroprotective effects of olive oil or its components were proven in various neurological disorders. A PREDIMED study (large randomised controlled-trial) recommended diet rich in polyphenols such as olive oil to improve cognitive functions even among the older population [10]. Another randomised control study reported 30% reduced risk of having a heart attack, stroke or death due to cardiovascular events following the consumption of olive oil [11]. In addition, phenolic-rich extra virgin olive oil (H-EVOO) treatment upregulated the expression of genes associated with synaptic plasticity such as Notch1, bone morphogenic protein (BMP), nerve growth factor receptor (NGFR), glucagon like peptide 1 receptor (GLP1R), lysine acetyltransferase 6A (Myst3) and CREB regulated transcription coactivator 3 (CRTC3) in aged mice [12].

The cardio- and neuro-protective effects of olive oil are largely due to one of its major phenolic compounds, hydroxytyrosol (HT), that was reported to possess antioxidant, anti-cancer, anti-inflammatory and neuroprotective properties [9,13,14,15]. In a hypoxic-reoxygenation rodent model, HT attenuated damage to the brain tissue by reducing the lactate dehydrogenase efflux [13]. Moreover, the neuroprotective effects of HT were also documented in diabetic neuropathy, where the compound significantly improved thermal response latency, which indicates the protection of small sensory fibers against diabetes [14]. Apart from its neuroprotective effects, HT also was shown to have mitogenic effects on human osteoblastic and umbilical vein endothelial cells [16,17].

Peripheral axonal regeneration depends on the presence of its supportive counterparts, the Schwann cells (SC) [18]. SCs can determine the survival of injured neurons by continuously secreting factors to promote axonal growth from the proximal end towards the distal stump [19].

To date, the potential neuroprotective effects of HT in the peripheral nervous system, especially towards the SC population, is poorly explored. In the present study, we seek to investigate the direct effects of HT on the proliferation of the human Schwann cell (hSC) using in vitro culture.

## 2. Results

### 2.1. Characterization of hSC

**Figure 1:**
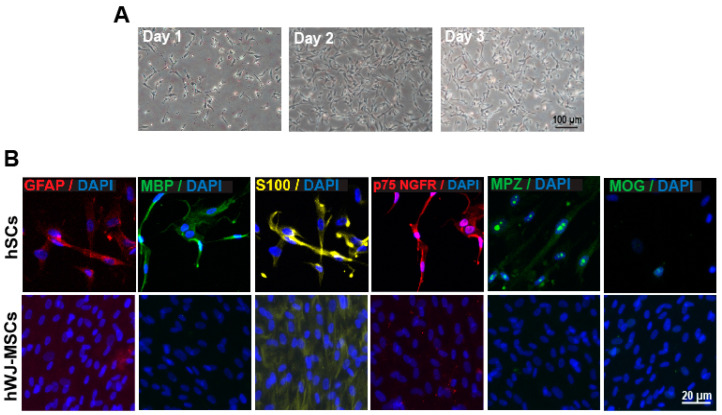
(**A**) **Morphology of human Schwann cell (hSC) in culture.** hSCs were cultured, maintained and assumed a spindle, bipolar shape after 4 days. (**B**) **Characterization of hSC through immunocytochemistry.** Cultures were immunostained with antibodies against p75 NGFR, GFAP, MPZ, s100, MBP, MOG as indicated in the figure. hSCs do not express MOG. All cultures were counterstained with 4′,6-diamidino-2-phenylindole (DAPI) (blue). Human Wharton jelly mesenchymal stem cells (hWJ-MSCs) were used as the negative control.

hSCs at passage 4–6 were cultured for 3–4 days under an optimized culture environment. Figure 1A illustrates the phase-contrast images of hSCs in culture from day 1 to day 3. The majority of hSCs were in spindle-like shape, bipolar and some were polygonal. The hSCs relatively proliferate well in the hSC culture medium.

To ensure the culture consisted of only hSC population, the hSCs were further characterized by immunostaining with p 75 nerve growth factor receptor (p75 NGFR), s100 protein subunit beta (s100β), glial fibrillary acidic protein (GFAP), myelin basic protein (MBP), myelin protein zero (MPZ) and myelin oligodendrocyte glycoprotein (MOG). The hSCs were positively stained with the specific marker for hSC (p75 NGFR, s100, GFAP, MBP, and MPZ) and negatively stained with MOG, a specific marker for oligodendrocyte (Figure 1B).

### 2.2. Low-Dose HT Increased the Viability and Proliferation of hSC

**Figure 2:**
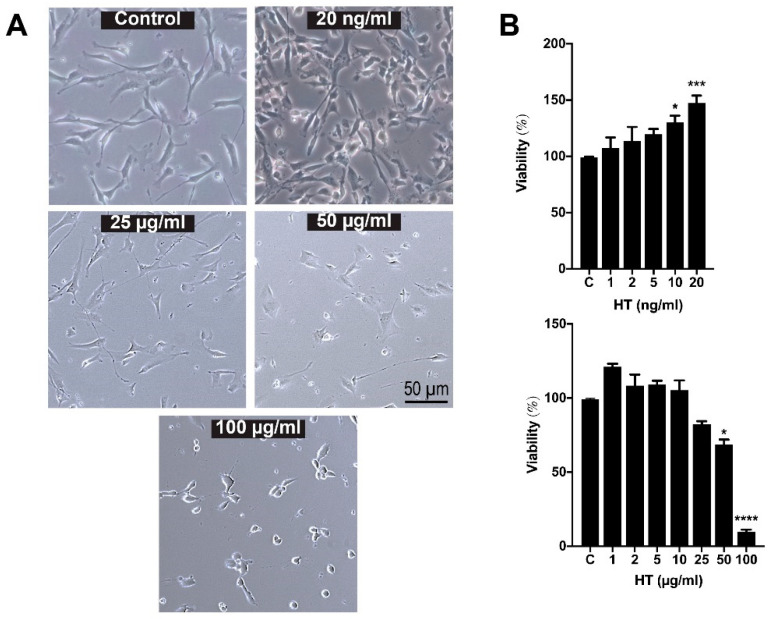
(**A**) **Morphology of hSC after 24-h treatment with hydroxytyrosol (HT).** Hydroxytyrosol does not alter the morphology of hSCs after 24 h of treatment, except at concentrations of 50 and 100 µg/mL whereby hSCs started to shrink. (**B**) **Cell viability assay of hSC after 24 h of treatment with HT.** One-way ANOVA and post hoc Tukey test revealed a significant increase in hSC proliferation following treatment with HT at concentrations of 10 and 20 ng/mL when compared to the control group. HT is significantly toxic to hSCs at 50 and 100 µg/mL. *p*-value * < 0.05, *** < 0.001, **** < 0.0001 vs. control.

To assess the effective dose response of HT, hSCs were treated with various concentrations of HT for 24 h. As shown in Figure 2A, HT did not alter the morphology of hSCs after 24 h of treatment, except at concentrations of 50 and 100 µg/mL where the hSCs started to become round and shrunk. The effect of HT treatments at different concentrations on the proliferation of hSCs was determined by MTT assay as shown in Figure 2B. One-way ANOVA analysis revealed a significant effect of treatment (F (13,41) = 23.01; *p* < 0.0001). The treatment of HT at 10 (130.12 ± 5.9%) and 20 ng/mL (147.8 ± 6.7%) significantly increased the cell number while maintaining cell viability of hSCs compared to the untreated cells (control) (*p*-value < 0.05) (Figure 2B). The higher concentrations of HT, 50 (68.5 ± 3.4%) and 100 µg/mL (28.3 ± 7.1%), profoundly reduced the viability of the hSCs (*p*-value < 0.05). Therefore, HT at 20 ng/mL was chosen to be used in the subsequent experiments and described as “HT” in the ensuing text.

HT at low doses (10 and 20 ng/mL) promotes proliferation, whereas at high doses (50 and 100 µg/mL) it inhibits proliferation. A similar trend in the effects of HT was reported previously where HT at low concentration promotes proliferation of human osteoblastic (1 × 10^−5^–10^−7^ M) and umbilical vein endothelial cells (30 µm) [16,17]. High dose of HT was tested for anti-cancer properties in thyroid [20], gastroenterology [21,22,23], and breast cancer [24] where high-dose HT inhibits cell proliferation and induces cell apoptosis. Therefore, HT at 20 ng/mL was chosen to be used in the subsequent experiments and is described as “HT” in the ensuing text.

### 2.3. HT Increased the Population of Proliferating hSC

**Figure 3:**
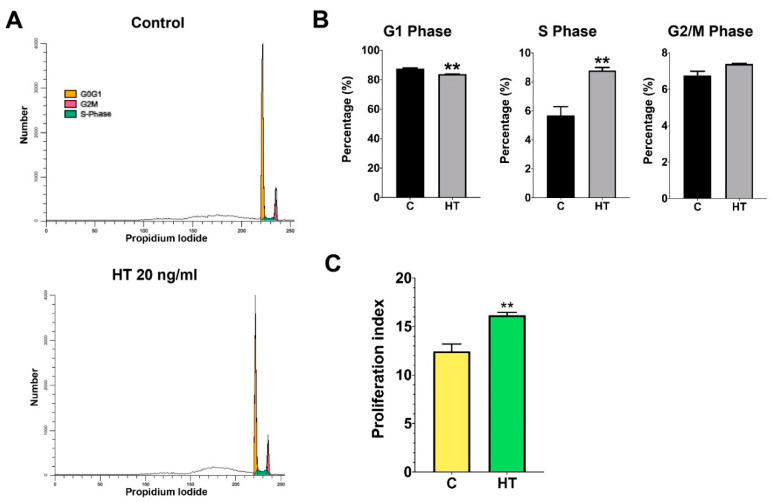
(**A**) Histogram representing the distribution of hSCs supplemented with HT at different stages of the cell cycle. Treatment of HT allows normal cell cycle progression of hSCs. (**B**) Percentage of cell population at different phases of the cell cycle. Quantification of the cell cycle distribution and the percentage of the distinct cell cycle phases in hSCs treated with HT were assessed using the ModFit software. An independent t-test was conducted to measure the significant difference between the HT-treated group and the control group (*p*-value ** < 0.01). (**C**) Proliferation index of HT-treated hSCs in comparison to control group.

HT treatment allowed normal cell cycle progression in hSCs (Figure 3A). Cell cycle analysis demonstrated that HT-treated hSCs have a lower percentage of cells in the G1 phase (t (4) = 7.77, *p* = 0.0015) than in the control group (Figure 3B). In the G2/M phase, no significant changes were observed between the HT-treated group and the control group. However, we found a significantly higher S-phase percentage (8.8 ± 0.2%) in the HT group when compared to the control (5.7 ± 0.6%), (t (4) = 4.78, *p* = 0.0088), respectively. The proliferation index (PI = S + G2/M) of HT-treated hSCs was significantly higher than that of the control group (t (4) = 7.76, *p* = 0.0015), indicating that HT increased DNA synthesis, subsequently resulting in escalated cell proliferation (Figure 3C).

Cell cycle analysis is a good indicator to demonstrate cell proliferation [25]. The S-phase represents the starting point of DNA replication (cells committed to proliferate) and is followed by the G2 phase where protein synthesis and cell growth take place before the cell enters the mitotic phase (M phase) [25].

### 2.4. HT Increased the Expression of Proliferative Protein Markers of hSC

**Figure 4:**
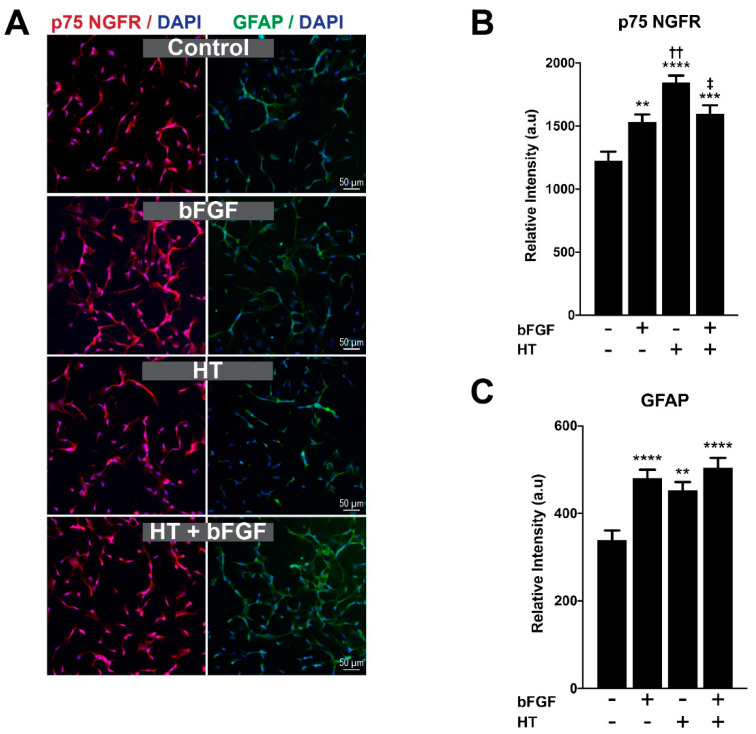
(**A**) **Immunostaining of hSC with p 75 NGFR and GFAP.** Schwann cells were labelled with p75 NGFR (red) and GFAP (green) in four different groups. Nuclei were counterstained with DAPI (blue) in all images. (**B**) **Expression of p75 NGFR and** (**C**) **GFAP.** Hydroxytyrosol significantly increased the protein expression of p75 NGFR and GFAP compared to control. *p*-value ** < 0.01, *** < 0.001, **** < 0.0001 vs. control; *p*-value †† < 0.01 vs. bFGF; *p*-value ‡ < 0.05 vs. HT. Data were analyzed with one-way ANOVA and post hoc Tukey’s test.

The differentiation of SC to its mature form can be observed through the expression of specific proteins at distinct SC developmental stages [26]. Immature SCs express proteins such as p75 NGFR and GFAP that are commonly expressed during SCs’ proliferative stage and downregulated in more mature stages. In this study, GFAP and p75 NGFR were employed as proliferative markers of hSC, which were measured after 48 h through immunocytochemistry and fluorescence image quantification (Figure 4A). Basic fibroblast growth factor (bFGF) was incorporated in the experiment as the positive control for the proliferation of hSCs. The bFGF is widely known for its role as the mitogenic factor to numerous types of cells, including, SCs [27]. One-way ANOVA showed a significant effect of treatment towards the expression of p75 NGFR (F (3,299) = 15.77; *p* < 0.0001). Figure 4B reveals a significant increase in the expression of p75 NGFR across all treatment groups, bFGF (1532.7 ± 58.7 a.u), HT (1844.0 ± 56.7 a.u) and bFGF + HT (1595.9 ± 69.5 a.u) when compared to the control group (1225.5 ± 70.5 a.u) (*p*-value < 0.05). HT-treated hSCs have a significantly higher expression of p75 NGFR compared to those in the bFGF (*p*-value < 0.01), and bFGF + HT (*p*-value < 0.05) groups.

Similarly, GFAP also markedly increased in all treatment groups (F (3,235) = 10.10; *p* < 0.0001). Post hoc analysis revealed a profound increase in the bFGF (480.8 ± 18.7 a.u), HT (452.3 ± 18.9 a.u), and bFGF + HT groups (504.3 ± 22.4 a.u) compared to the control group (339.2 ± 21.7 a.u) (*p*-value < 0.05) (Figure 4C). No significant difference in GFAP expression was observed among the treatment groups (*p* > 0.05).

The synergistic effect of bFGF and HT combinations can be evaluated through the calculation of the coefficient of drug interaction (CDI) by the following equation: CDI = AB/(A B), where AB—relative protein expression of the combination (bFGF + HT); A or B—relative protein expression of the single treatment (bFGF or HT). A coefficient of drug interaction < 1 indicates a synergistic effect; CDI = 1 indicates an additive effect; CDI > 1 indicates an antagonistic effect [28,29,30]. Through this calculation, the effect of bFGF and HT was synergistic; CDI = 0.71 (effect on p75 NGFR expression) and CDI = 0.78 (effect on GFAP expression).

The mitogenic properties of bFGF and HT were reflected through their incremental effects on proliferation markers, such as GFAP and p75 NGFR. There was a profound increase in p75 NGFR expression in the HT group compared to bFGF, which indicates the greater potency of HT than bFGF as a mitogen for cell division. Although the effect of both substances was deemed synergistic through CDI calculation, the combination of bFGF + HT treatment significantly reduced the expression of p75 when compared to HT treatment alone.

## 3. Discussion

In this report, we have demonstrated the proliferative potential of HT in hSCs by (1) an increased proliferation of hSCs in MTT assay, (2) a higher proliferation index in cell cycle analysis and (3) an increased expression of hSC proliferative phenotype markers, including p75 NGFR and GFAP. To our knowledge, this is the first study that reports the effect of HT on hSC proliferation.

Anti-cancer activities of HT were reported in the past studies as high doses of HT inhibited cancer cells’ proliferation and induced their apoptotic cell death [20,21,22,23,24]. Nonetheless, in this current study, we report that HT is proliferative at a low dose and antiproliferative at a high dose (Figure 2B). In line with our findings, two previous studies reported the proliferative effects of HT in human osteoblastic and umbilical vein endothelial cells [16,17]. At this stage, we are unsure of reason for the opposing effects of extreme (low vs. high) doses of HT on the proliferation of hSCs, hence we are looking into these aspects in future studies.

It is still unclear how HT affects the proliferation of hSCs. The activation of the phosphoinositide 3-kinase PI3K/Akt pathway is deemed essential for the proliferation of SC, as enhanced activation of Akt accelerates G1-S progression of SC [31]. Cheng and colleagues reported that HT increased the expression of Akt, TGF-β and mTOR along the increased proliferation of human umbilical vein endothelial cells [16]. Another report indicated that HT activated PI3K/Akt pathways through secondary generation of reactive oxygen species that acted as the second messenger for its activation [32]. Therefore, the high proliferation index (S + G2/M) of HT-treated cells in this current study may be mediated by Akt-related pathways.

SCs are originally derived from the neural crest, a transient and multipotent embryonic structure that also generates other glial subtypes of the peripheral nervous system (PNS) [33,34]. During early embryonic phases, neural crest cells develop into Schwann cell precursors, which represents the first transitional stage of the Schwann cell lineage. This is followed by differentiation into a proliferative phenotype called immature SCs [33]. The presence of the immature SCs is identified by the expression of distinct proteins, including neural cell adhesion molecule (NCAM), L1 cell adhesion molecule (L1CAM), growth associated protein 43 (GAP-43), A5E3, Ran-2, GFAP and p75 NGFR [33]. These proteins are markedly down-regulated during the transition of immature SC to myelinating type [33]. Our findings showed that HT significantly increased the proliferation of hSCs, as indicated by the increased expression of GFAP and p75 NGFR.

GFAP and p75 NGFR are specifically found in the proliferative phenotype of SC (immature SC). The p75 NGFR, which belongs to the tumor necrosis factor (TNF)-receptor superfamily, is involved in several actions of SC, particularly SC migration, proliferation and myelination [35]. On the other hand, GFAP is an intermediate filament protein that is responsible for the cytoskeletal structure of most glial cells, as it preserves their mechanical strength and supports neighboring neurons [36]. Loss of these proteins as exemplified by the p75 NGFR (−/−) and GFAP (−/−) knockout mice impaired SC proliferation and motor recovery of the transected nerve [37,38]. Thus, based on the existing literature and our findings, p75 NGFR and GFAP are vital for SCs’ proliferation, and their expressions in immature SCs are indicative of proliferative phenotypes.

Basic fibroblast growth factor was first identified to be mitogenic by Davis and Stroobant [39]. Exogenous application of bFGF was also reported to promote neurite extension and vascularization of the regenerating nerve fibers in the transected sciatic nerve in rats [40]. The bFGF mediates its action, mostly through ligand binding to the FGF receptor that triggers the mitogenic cascades in most cells. As illustrated in Figure 4B, bFGF simultaneously increased the expression of p75 NGFR and GFAP. In line with our findings, previous studies have reported bFGF to increase the gene and protein expression of p75 NGFR in the human neuroblastoma cell line and expressions of GFAP mRNA in astrocytes [41,42].

There are several limitations in our study. First, our laboratory investigation was conducted in vitro using SCs culture alone, hence the effects of HT observed in this study should not yet be extrapolated to the treatment of human nerve injuries. The main aim of this research was to demonstrate the direct effect of HT on SCs. Therefore, we employed a SC culture alone in this project. At this point, we can speculate that HT at 20 ng/mL has the potential to increase the proliferation of SCs. However, we are yet to prove whether the same dosage or dosage range may have similar effects in more complex biological environments such as a SC-neuronal co-culture or in vivo models.

## 4. Materials and Methods

### 4.1. Experimental Design

This study is an invitro design that intends to evaluate the effects of HT on the proliferative capability of a primary hSC culture. The effective dose of HT was selected based on the highest cell viability indicated by the effects of HT in the MTT assay. For protein analysis, the SC culture was grouped as (a) control (SC culture only), (b) SC + HT (effective dose), (c) SC + bFGF (10 ng/mL), (d) SC + bFGF (10 ng/mL) + HT (effective dose).

### 4.2. Preparation of Primary hSC Culture

#### 4.2.1. Vessel Coating

For adherence of hSC to the culture vessel, the vessel was coated firstly with poly-l-lysine prepared by adding 10 mL of sterile water and 15 uL of poly-l-lysine stock solution (1 mg/mL) (ScienCell Research Laboratories, Carlsbad, CA, USA) to the vessel. The vessel was left in a 37 °C with 5% carbon dioxide (CO_2_) incubator for an hour. After the incubation, the poly-l-lysine coated vessel was rinsed with sterile water twice and then 15 mL of hSC medium (ScienCell Research Laboratories, USA) with hSC suspension was added.

#### 4.2.2. Cell Culture

The hSC was purchased (ScienCell Research Laboratories, USA) and maintained in the hSC medium. An hSC cryovial containing > 5 × 10^5^ cells in 1 mL volume were thawed and suspended in 15 mL of hSC medium. The suspension was seeded in a culture vessel and left for one-day incubation at 37 °C. The culture medium was changed the next day to remove residual dimethyl sulfoxide (DMSO) and unattached cells, then every other day thereafter. All cells were maintained in the culture at 37 °C in an atmosphere of 5% CO_2_. The medium was replaced every two days.

### 4.3. Proliferation Assay

The effect of HT on hSC proliferation (Sigma, St. Louis, MO, USA) was evaluated using the Vybrant™ MTT (3-(4,5-dimethylthiazol-2-yl)-2,5-diphenyltetrazolium bromide) cell proliferation assay kit (Invitrogen (Thermo-Fisher Scientific), Carlsbad, CA, USA) following the manufacturer’s recommendations. Briefly, the hSC culture at passage 4–6 was cultured in triplicate in a 96-well plate at a density of 10,000 cells/cm^2^ in the culture medium containing 10% FBS for one to two days. Then, the medium was changed and the cells were subjected to various concentrations of HT. The MTT assay was carried out after 24-h post-treatment. During the assay, 100 μL of MTT solution was added and incubated with the cells for 4 h. Then, the cells were lysed with DMSO to release and solubilize the purple formazan. The absorbance value of the culture in each well was measured at a wavelength of 570 nm as per the manufacturer’s instruction.

### 4.4. Immunocytochemical Analysis

After 48 h with a seeding density of 10,000 cells/cm^2^ of the hSC culture at passage 4–6, all samples were fixed with 4% paraformaldehyde (PFA) for 15 min at room temperature and washed thrice with PBS. The fixed samples were then soaked in PBS containing 0.5% Triton X-100 (Sigma, USA), followed by three times washing with PBS. Next, the samples were incubated with 10% goat serum (Gibco^®^ (Thermo Fisher Scientific), Carlsbad, CA, USA) at 37 °C for 1 h. The goat serum was removed and the samples were loaded with primary antibodies, which include anti-myelin basic protein (MBP, 1:200, Invitrogen), anti-myelin protein zero (MPZ, 1:200, Avivasysbio, San Diego, CA, USA), anti-glial fibrillary acidic protein (GFAP, 3 µg/mL, STEMCell Technologies, Vancouver, Canada), anti-p75 nerve growth factor receptor (p75 NGFR, 1:200, Abcam, Cambridge, MA, USA), anti-s100β (1:200, Abcam) and anti-myelin oligodendrocyte glycoprotein (MOG, 3 µg/mL, Abcam). All samples with primary antibodies were incubated in Dulbecco’s phosphate-buffered saline (DPBS) at 4 °C overnight. The samples were then washed with PBS containing 0.1% Tween-20 thrice and incubated with Alexa Fluor 594 anti-rabbit IgG (1:300, Invitrogen, USA) and Alexa Fluor 488 anti-mouse (1:300, Invitrogen, USA) for 1 h at 37 °C. The nuclei of cells were counterstained with 4′,6-diamidino-2-phenylindole (DAPI) (Life Technologies (Thermo-Fisher Scientific), Carlsbad, CA, USA, 1:15000) for 15 min at room temperature. The culture was visualized under a fluorescence microscope (Nikon A1, Nikon Corporation, Tokyo, Japan). The images were recolored and edited with Adobe Photoshop CC (Version 2017.1.1, San Jose, CA, USA).

### 4.5. Image Quantification for Protein Expression

The protein expression of hSC was determined by measuring the relative mean fluorescence intensity from the immunofluorescence images. For each sample, a minimum of five different independent fields of images in each biological replicate were captured. The exposure time and hardware gain of the fluorescence imaging were constantly maintained throughout all samples for each respective marker. These images were then quantified through an automated cell region of interest (ROI) labelling and the intensity of the individual cells selected was computed and analyzed using NIS-Elements D image-processing software (Nikon NIS-Elements D Ver4.3, Japan). The relative mean intensity was determined and expressed as arbitrary units (a.u).

### 4.6. Cell Cycle Analysis

The hSC culture was processed using CycleTEST PLUS DNA Reagent Kit (Becton Dickinson, New Jersey, USA) according to the manufacturer’s instruction. In brief, passage 4–6 of hSCs were cultured separately in an untreated and treated medium with HT. After reaching 95% confluency, the cells were detached from the culture surface by trypsinization followed by centrifugation at 1400 rpm for 5 min. The pellet was then resuspended in 1 mL of the buffer solution. A total number of 1 × 10^6^ cells per sample were used. Cells were then stained with propidium iodide (PI). PI-stained single nuclei suspensions were analyzed using FACS Calibur flow cytometer (Becton Dickinson, USA), and raw data were collected using CELLQuest software (Becton Dickinson, USA). Data analysis was performed using Modfit Cell Cycle Analysis Software (Verity House Software, Topsham, ME, USA).

### 4.7. Statistical Analysis

Data analysis was completed using Graphpad Prism 8 (Version 8.2.1, San Diego, CA, USA). Average data are shown as the mean  ±  standard error of mean (SEM). One-way analysis of variance (ANOVA) was used to analyze the data for cell viability (MTT assay) and protein expression. The differences between individual means were compared with a post hoc Tukey’s test and statistical significance was set at a value of *p*  <  0.05. The data for the cell cycle were analyzed by an independent t-test.

## 5. Conclusions

HT at 20 ng/mL stimulates the proliferation of hSCs in vitro, indicated by the significant increase in hSC proliferation index and protein expression of hSCs’ proliferation markers, namely p75 NGFR and GFAP.
